# Humoral Response to mRNA-1273 SARS-CoV-2 Vaccine in Peritoneal Dialysis Patients: Is Boostering After Six Months Adequate?

**DOI:** 10.3389/fmed.2022.905798

**Published:** 2022-06-24

**Authors:** Georg Beilhack, Rossella Monteforte, Florian Frommlet, Roman Reindl-Schwaighofer, Robert Strassl, Andreas Vychytil

**Affiliations:** ^1^Division of Nephrology and Dialysis, Department of Medicine III, Medical University of Vienna, Vienna, Austria; ^2^Center for Medical Statistics, Informatics and Intelligent Systems, Medical University of Vienna, Vienna, Austria; ^3^Division of Clinical Virology, Medical University of Vienna, Vienna, Austria

**Keywords:** COVID-19, peritoneal dialysis, anti-SARS-CoV-2 antibodies, booster, mRNA-1273 vaccine, spikevax

## Abstract

In dialysis patients the humoral response to anti-SARS-CoV-2 vaccines is attenuated and rapidly declines over time. However, data on the persistence of the immune response in peritoneal dialysis (PD) patients are scarce, particularly after a third (booster) dose with mRNA-1273 vaccine. In this prospective cohort study, we report anti-SARS-CoV-2 antibody levels in PD patients before and after the third dose of mRNA-1273 vaccine. Six months after the second dose, anti-SARS-CoV-2 antibodies were detected in all patients (*n* = 34). However, within this time period antibodies substantially declined in 31 of 34 patients (4.5-fold, median = 192 BAU/mL, *p* = 1.27 × 10^–9^) and increased in three patients. In accordance with government regulations, a third dose of mRNA-1273 vaccine (50 μg) was given to 27 PD patients 6 months after the second dose which induced a significant increase of anti-SARS-CoV-2 antibody titers (58.6-fold, median = 19405 BAU/mL, *p* = 1.24 × 10^–29^). A mixed model analysis showed that a lower Davies Comorbidity Score and a higher GFR were associated with higher antibody titers (*p* = 0.03 and *p* = 0.02). The most common adverse events after the third dose were pain at the injection site (77.8%) and fatigue (51.9%). No hospitalizations were reported. In conclusion, 6 months after the second dose of mRNA-1273 vaccine, anti-SARS-CoV-2 antibodies substantially decreased in PD patients, whereas a well-tolerated third dose induced a robust humoral response. Our data suggest that the administration of a booster dose within a shorter interval than 6 months should be considered in PD patients in order to maintain high anti-SARS-CoV-2 antibody levels and assure protection from severe COVID-19 disease.

## Introduction

In November 2021, the Austrian authorities recommended a third vaccine dose in order to prevent severe acute respiratory syndrome caused by coronavirus 2 (SARS-CoV-2) in the adult population and to contain the fourth wave of coronavirus disease 2019 (COVID-19) due to the B1.6.17.2/Delta variant. This decision was supported by data on significant waning of anti-SARS-CoV-2 antibodies in adults within 6 months from the second vaccine dose (1, 2). Several studies reported a decreased immune response to two doses of COVID-19 vaccines in dialysis patients compared to the general population and a substantial loss of anti-SARS-CoV-2 antibodies over time (3–6). The majority of these studies focused on hemodialysis (HD) patients receiving the BNTb162b2 vaccine and only few on peritoneal dialysis (PD) patients. Our recently published data on the early immune response to the mRNA-1273 SARS-CoV-2 vaccine in PD patients showed a high seroconversion rate (97.4%) and high antibody levels after two doses (7).

Here, we report anti-SARS-CoV-2 S antibody levels in PD patients 6 months after the second dose and 1 month after the third dose of the mRNA-1273 vaccine. We assessed factors potentially associated with the humoral response and report adverse events (AEs) to the third vaccine dose.

## Patients and Methods

### Study Population

This prospective single-center cohort study was performed at the Division of Nephrology and Dialysis, Department of Medicine III, Medical University of Vienna, Austria. We included adult PD patients who had received two doses of mRNA-1273 vaccine against SARS-CoV-2. Patients with previous or active COVID-19 infection were excluded. All patients attended our outpatient clinics on a regular basis, where they were tested for COVID-19 infection (PCR) and were checked for SARS-CoV-2 specific symptoms. The cohort (*n* = 39 at baseline) received the first dose (100 μg mRNA-1273) on March 11th, 2021 and the second dose (100 μg mRNA-1273) on April 8th, 2021. These retrospective data were published recently (7). In the present prospective study we measured anti-SARS-CoV-2 S antibody levels 6 months after the second dose and 1 month after the third dose (50 μg mRNA-1273). Comorbidities of our PD patients were evaluated using the co-morbidity score published by Davies et al. (8).

### Serological Assessment

Blood was drawn from patients as part of their routine visits 6 months after the second dose and 1 month after the third dose, respectively. The antibody response against SARS-CoV-2 S was measured using the Roche Elecsys anti-SARS-CoV-2 S^®^ assay on a Roche Cobas e801 platform according to manufacturer instructions (ROCHE^®^ Diagnostics International Ltd.). This assay detects antibodies against the receptor binding domain (RBD) of the SARS-CoV-2 spike (S) protein. RBD-targeting antibodies have been described to have a high neutralization activity against SARS-CoV-2 (9). The detection range of the assay is between 0.4 BAU/mL and 250 BAU/mL. Patient sera with antibody concentrations above the upper detection limit were further diluted. The new upper detection limit was 25,000 BAU/mL. The assay was calibrated to the current WHO International Standard for antibody detection against SARS-CoV-2 S. To detect whether asymptomatic patients had a previous infection with the SARS-CoV-2 virus, a serological assay (ROCHE^®^ Elecsys anti-SARS-CoV-2 assay) was used to measure antibodies targeting the nucleocapsid (N) antigen (1 month and 6 months after the second vaccine dose). Patients testing positive for anti-nucleocapsid (N) antibodies were excluded from the study.

### Reporting of Adverse Events

Adverse events (AEs) occurring within 7 days from the third vaccination were recorded using a standardized survey. AEs were categorized in local AEs (pain, swelling, bruising) and systemic AEs (fever, headache, fatigue, muscle pain, joint pain, dizziness, vomiting). The patients were asked to grade their AEs using a scale from 0 to 4 (Grade = 0: no event; grade = 1: mild, does not affect daily activities; grade = 2: moderate, interferes with activities of daily living; grade = 3: severe, interrupts usual activities of daily living; grade = 4: hospitalization).

### Statistical Analysis

Descriptive statistics are given as mean and standard deviation or median and interquartile range, as appropriate. Statistical analysis was performed with R version 4.1.1. A mixed model was fitted for the logarithm of antibody levels as outcome variable, a random intercept for each patient and time as fixed effect, where the four different time points were considered as a factor variable. Tests were performed for the contrasts between adjacent time points, that is differences in antibody levels before and after the second vaccination, after second vaccination and 6 months later, 6 months after second vaccination and after booster shot. Mixed model analysis was performed with the R packages lme4 (10) and lmerTest (11). To assess the potential influence of other covariates on antibody levels the following variables were considered as fixed effects, respectively, in a mixed model which again had patient as random effect: age, gender, albumin, dialysis vintage, vitamin D, ferritin, GFR and Davies Comorbidity Score. To illustrate the underlying effects for statistically significant covariates scatterplots with linear models fitted for each time point are provided.

Since this is a single center study, the sample size (*n* = 39) was limited by the number of patients treated with peritoneal dialysis in the PD unit of the Medical University of Vienna.

### Ethical Considerations

Our study was approved by the ethics committee of the Medical University of Vienna (EK1362/2020). Written informed consent was obtained from all patients. All participants were informed about their antibody levels. Furthermore, we explained that the exact value of antibody titers which protects from COVID-19 infection is currently unknown. Patients were advised to continue keeping safety measures such as social distancing, hygiene standards and face masks after vaccination.

## Results

**FIGURE 1:**
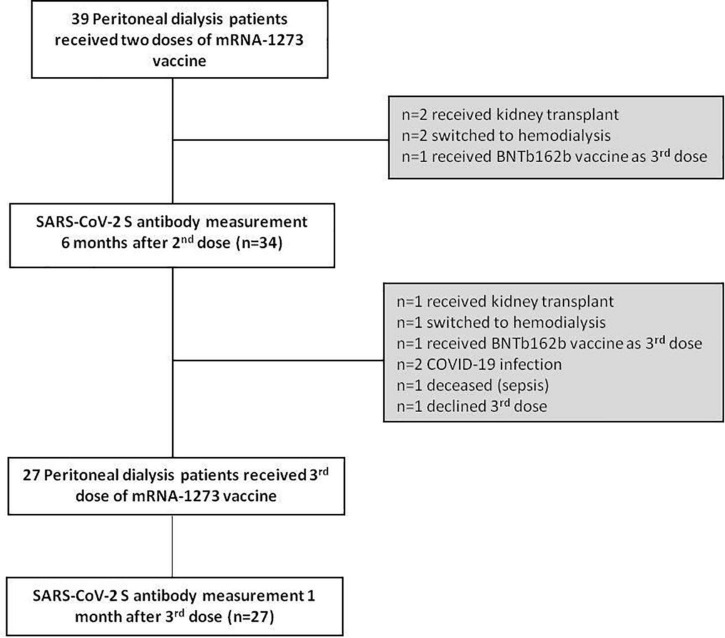
Study flowchart of the peritoneal dialysis patient cohort.

A patient flow diagram and detailed characteristics of the study cohort are shown in Figure 1 and Table 1. Of the 39 patients at baseline, 5 were excluded (*n* = 2 received kidney transplant, *n* = 2 switched to hemodialysis and *n* = 1 received BNTb162b as third dose). Therefore, 6 months after the second dose of mRNA-1273 vaccine we prospectively measured anti-SARS-CoV-2 S antibody levels in 34 patients (22 men, 12 women). The mean age was 54.1 years (range 29–77 years) with a median dialysis vintage of 14.5 months (IQR 4.8–30.3 months). Within this period of 6 months after the second dose no COVID-19 infection occurred among these patients and none of the patients was tested positive for anti-nucleocapsid (N) antibodies.

**TABLE 1:** Characteristics of peritoneal dialysis patients vaccinated with mRNA-1273 vaccine.

Patient demographics	Baseline	6 months after 2nd dose	Received 3rd dose
Total number of patients included	39	34	27
Age (years, mean, range)	55.2 (29–80)	54.1 (29–77)	54.3 (33–76)
Men (%)	26 (66.7)	22 (64.7)	17 (63)
**Primary kidney disease**			
Diabetes	11	10	7
Vascular disease	3	1	1
Glomerulonephritis	8	7	6
ADPKD	4	3	2
Unknown	5	5	4
Other	8	8	7
**Davies comorbidity score (%)**			
0	14 (36)	12 (35)	11 (41)
1	14 (36)	13 (38)	9 (33)
2	7 (18)	6 (18)	5 (19)
3	3 (7.7)	3 (9)	2 (7)
4	0	0	0
5	1 (2.6)	0	0
Dialysis vintage, months median (IQR)	16.1 (6–31.3)	14.5 (4.8–30.3)	13.4 (5.25–30.6)
Weekly total (renal + peritoneal) Kt/V median (IQR)	1.94 (1.78–2.35)	1.94 (1.67–2.16)	1.94 (1.77–2.17)
GFR* (mL/min) median (IQR)	1.95 (0.76–6.01)	1.59 (0.52–5.26)	1.57 (0.56–4.99)
**Blood Group AB0 (%)**			
0	19 (48.7)	18 (52.9)	13 (48.1)
A	12 (30.8)	9 (26.5)	8 (29.6)
B	6 (15.4)	6 (17.6)	1 (3.7)
AB	2 (5.1)	1 (2.9)	5 (18.5)
**Laboratory (mean ± SD)**			
Hemoglobin (g/dL)	10.6 ± 1.39	10.3 ± 1.43	10.4 ± 1.41
Leukocytes (G/L)	7.2 ± 2.11	7.6 ± 2.05	7.7 ± 1.98
Thrombocytes (G/L)	225 ± 75.34	231 ± 71.1	234 ± 76.5
Albumin (g/L)	3.6 ± 3.45	36.2 ± 5.08	36.7 ± 4.84
Sodium (mmol/L)	136 ± 3.23	136 ± 3.98	136 ± 3.8
Potassium (mmol/L)	4.4 ± 0.63	4.31 ± 0.46	4.3 ± 0.47
Calcium (mmol/L)	2.25 ± 0.18	2.21 ± 0.23	2.24 ± 0.21
Phosphate (mmol/L)	1.77 ± 0.42	1.81 ± 0.49	1.84 ± 0.48
Bicarbonate (mmol/L)	25.8 ± 3.25	25.9 ± 3.01	26.5 ± 2.91
C-reactive protein (mg/dL)	0.52 ± 0.69	0.97 ± 1.57	0.85 ± 1.52
Body mass index (kg/m^2^)	27 ± 5.37	26.6 ± 5.02	26 ± 4.69
Obesity (BMI > 30 kg/m^2^) (%)	10 (25.6)	6 (17.6)	3 (11.1)
**Medication (***n***)**			
Immunosuppressive therapy	7	5	5
RAAS-inhibitor	20	17	14
Vitamin D	32	25	18
**Antibody titer (BAU/mL) median (IQR)**			
1 month after 1st dose	6.62 (1.57–22.5)		
1 month after 2nd dose	968 (422.5–2244.5)		
6 months after 2nd dose		192 (84.5–685.0)	
1 month after 3rd dose			19,405 (8,884–25,000)

Data are presented as n (%), mean ± SD or as median (IQR); Other = Alport syndrome (1), GVHD-associated TMA (1), cardiorenal syndrome (1), secondary FSGS (1), AL-Amyloidosis (1), glomerulosclerosis (1), nephronophthisis (1), scleroderma (1); ADPKD, autosomal dominant polycystic kidney disease; GVHD, graft versus host disease; TMA, thrombotic microangiopathy; FSGS, focal segmental glomerulosclerosis; BMI, body mass index; weekly Kt/V, clearance of urea × time/volume; GFR, residual glomerular filtration rate; RAAS, renin-angiotensin-aldosteron system; CNI, calcineurin inhibitors; MMF, mycophenolate-mofetil. *residual GFR was calculated as mean of renal creatinine and renal urea clearance using 24 h urine samples.

**FIGURE 2:**
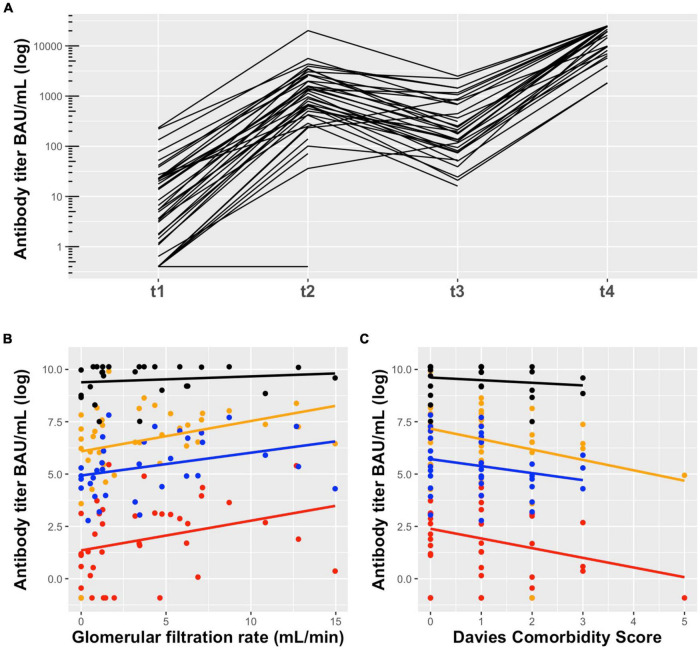
**(A)** Anti-SARS-CoV-2 S antibody levels in peritoneal dialysis patients in response to mRNA-1273 vaccine: t1 = 1 month after the first dose (median = 6.62 BAU/mL, IQR 1.57–22.50), t2 = 1 month after the second dose (median = 968 BAU/mL, IQR 422.50–2244.5), t3 = 6 months after the second dose (median = 192 BAU/mL, IQR 84.5–685.0), and t4 = 1 month after the third dose (median = 19,405 BAU/mL, IQR 8,884–25,000). Six months after the second dose antibodies substantially declined and increased significantly after the third dose of mRNA-1273 vaccine. The technical lower and upper limit of detection were 0.4 BAU/mL and 25,000 BAU/mL, respectively. **(B)** Association of GFR and **(C)** Davies Comorbidity Score with anti-SARS-CoV-2 S antibody titers in peritoneal dialysis patients in response to mRNA-1273 vaccine 1 month after the first dose (red), 1 month after the second dose (orange), 6 months after the second dose (blue) and 1 month after the third dose (black). A higher GFR and lower Davies comorbidity score are associated with higher antibody titers. This association was particularly evident after the first and second dose, but almost disappeared after the third dose.

Six months after the second dose, anti-SARS-CoV-2 S antibodies were detected in all patients (*n* = 34), although they substantially declined in 31 of 34 patients (91.2%) (4.5-fold decrease compared to 1 month after the second dose, median = 192 BAU/mL, IQR 84.5–685.0, *p* = 1.27 × 10^–9^) (Figure 2A). Three of 34 patients did not follow the common pattern of waning antibodies. These patients had a low humoral response after two doses of mRNA-1273 vaccine, but continuously increased their anti-SARS-CoV-2 S antibody levels during the observed time. All three patients had experienced kidney graft failure before PD start and two of them still received immunosuppressive therapy (Table 2).

**TABLE 2:** Antibody titers after mRNA-1273 vaccination in peritoneal dialysis patients with immunosuppressive therapy.

Sex, age	IS	Dosage	Indication	Antibody titer (BAU/mL) 1 month after 1st dose	Antibody titer (BAU/mL) 1 month after 2nd dose	Antibody titer (BAU/mL) 6 months after 2nd dose	Antibody titer (BAU/mL) 1 month after 3rd dose
M, 43	CNI	2 mg q.d.	Lung transplant	<0.40	<0.40	NA*	NA*
	Prednisolon	5 mg q.d.					
	MMF	250 mg t.i.d.					
M, 43	MMF	500 mg q.d.	Kidney graft failure	<0.40	234.00	447.00	16214.00
F, 35	MMF	500 mg b.i.d.	Kidney graft failure	27.10	250.00	878.00	25000.00
F, 74	CNI	0.5 mg q.d.	Kidney graft failure	17.70	1330.00	1165.00	25000.00
	Prednisolon	2.5 mg q.d.					
M, 54	Prednisolon	2.5 mg q.d.	Kidney graft failure	13.90	1543.00	825.00	9927.00
F, 55	Prednisolon	2.5 mg q.d.	Kidney graft failure	3.58	1962.00	254.00	19137.00
M, 76	Ustekinumab**	90 mg every 3 months	Psoriasis	20.00	677.00	39.10	19663.00

M, male; F, female; IS, immunosuppressive therapy; CNI, calcineurin inhibitor; MMF, mycophenolate-mofetil; <0.4 BAU/mL = negative (lower limit); 25,000 BAU/mL = maximum (upper limit); NA*, not available: this patient received a kidney transplant in July 2021 and was excluded from the study; **anti-IL-12/23.

Of the remaining 34 patients, 7 patients did not receive a third dose of mRNA-1273 while treated with PD (*n* = 1 received kidney transplant, *n* = 1 switched to hemodialysis, *n* = 1 received BNTb162b as third dose, *n* = 2 had COVID-19 infection, *n* = 1 died (sepsis), *n* = 1 declined the third dose). The two patients with COVID-19 disease (5.1%) were infected through family members in October 2021, before the third vaccine dose. In both cases no hospitalization was required, and these two patients could continue their PD treatment at home.

Twenty-seven PD patients (17 men, 10 women) received the third dose of mRNA-1273 vaccine (50 μg) on November 10th, 2021 (Figure 1 and Table 1). The mean age was 54.3 years (range 33–76 years) with a median dialysis vintage of 13.4 months (IQR 5.25–30.6 months). The third dose of the mRNA-1273 vaccine induced a significant increase of anti-SARS-CoV-2 S antibody levels in all patients (58.6-fold increase compared to 6 months after the second dose, median = 19,405 BAU/mL, IQR 8884–25,000, *p* = 1.24 × 10^–29^) (Figure 2A). Nine of 27 patients (30%) reached antibody levels of 25,000 BAU/mL (upper detection limit), among them were two patients with immunosuppressive therapy (Table 2).

In order to assess factors associated with the antibody response to mRNA-1273 vaccine, we performed a mixed model analysis for repeated measurements. This analysis indicated that a lower Davies Comorbidity Score and a higher GFR are associated with higher antibody levels (*p* = 0.03 and *p* = 0.02, respectively) (Figures 2B,C and Supplementary Table 1).

Patients were asked to report adverse events (AEs) to the third dose occurring within 1 week after vaccination. The most common adverse events (AEs) were pain at the injection site (77.8%) and fatigue (51.9%). All AEs were graded as mild, moderate or severe. No hospitalizations were reported (see Supplementary Figure 1).

## Discussion

In this study, we show a significant decrease of anti-SARS-CoV-2 S antibodies 6 months after two doses of mRNA-1273 vaccine in PD patients. Our data are in accordance with recently published studies describing waning anti-SARS-CoV-2 antibodies over time in the general population and in dialysis patients (1–5). This substantial decline of antibody levels was observed in response to vector- as well as mRNA-based anti-SARS-CoV-2 vaccines (6).

In our study, the third dose of mRNA-1273 vaccine led to a strong humoral response in all patients, even in patients with low antibody levels after the second dose. This observation is supported by recently published data showing a substantial increase of anti-SARS-CoV-2 antibodies after the third dose of BNT162b2, particularly in HD patients with low antibody levels after the second dose (12–14).

So far, few studies investigated factors associated with the immune response to the third dose of anti-SARS-CoV-2 vaccines in dialysis patients (12, 15, 16). These studies described more advanced age, immunosuppressive therapy, hematologic malignancies and lower gamma globulin concentration as potential factors with negative impact on humoral response. In our prior study (7) we found that a shorter dialysis vintage and lower Davies Comorbidity Score were correlated with higher antibody levels after the first mRNA-1273 dose. In the current study, we confirmed that a lower Davies Comorbidity Score correlates to higher antibody levels. In accordance with recently published data on patients after kidney transplantation (17) and those with diabetes (18), our mixed model analysis for repeated measurement identified a higher GFR to be associated with higher antibody levels in PD patients. Impairment of kidney function as well as the presence of co-morbidities have been reported as factors altering the immune system and influencing immune responses to vaccines and pathogens (3, 19).

The frequency and severity of the AEs after the third dose were comparable to the AEs observed in response to the second dose. This is in consistency with recently published data on dialysis patients using the BNT162b2 vaccine (12, 13).

A limitation of our study is the fairly small sample size due to the single center study design. We did not find a significant association between age and humoral response to vaccination, as suggested by some previous studies (12, 16). However, because of the young age of included patients compared to other study populations a relationship between advanced age and lower levels of anti-SARS-CoV-2 antibodies cannot be excluded. Further limitations include the lack of a control group and of cellular immunity data.

In conclusion, we report data of a well-characterized cohort of PD patients who received a third dose of mRNA-1273 vaccine in the absence of previous COVID-19 infection. Six months after the second dose, anti-SARS-CoV-2 antibodies substantially decreased, whereas a well-tolerated third dose induced a robust humoral response. Factors associated with improved antibody responses included a higher GFR and lower Davies Comorbidity Score. Since recent data indicate that higher antibody titers after vaccination correlate with a higher vaccine efficacy (20), we suggest that the administration of a booster dose within a shorter interval than 6 months should be considered in PD patients in order to maintain high anti-SARS-CoV-2 antibody levels and assure protection from severe COVID-19 disease.

## Data Availability Statement

The raw data supporting the conclusions of this article will be made available by the authors, without undue reservation.

## Ethics Statement

The studies involving human participants were reviewed and approved by the Ethics Committee of the Medical University of Vienna (EK1362/2020). The patients/participants provided their written informed consent to participate in this study.

## Author Contributions

GB contributed to the conception and design of the study, analyzed the data, and wrote the manuscript. RM analyzed the data and wrote sections of the manuscript. FF contributed to the design of the study and performed the statistical analysis. RR-S contributed to the conception and design of the study. RS performed antibody titer measurements. AV contributed to the conception and design of the study and wrote the manuscript. All authors contributed to the manuscript revision, read, and approved the submitted version.

## Conflict of Interest

The authors declare that the research was conducted in the absence of any commercial or financial relationships that could be construed as a potential conflict of interest.

## Publisher’s Note

All claims expressed in this article are solely those of the authors and do not necessarily represent those of their affiliated organizations, or those of the publisher, the editors and the reviewers. Any product that may be evaluated in this article, or claim that may be made by its manufacturer, is not guaranteed or endorsed by the publisher.

## Abbreviations

SARS-CoV-2: severe acute respiratory syndrome coronavirus 2
COVID-19: coronavirus disease 2019
HD: hemodialysis
PD: peritoneal dialysis
BAU: binding antibody units
AEs: adverse events
MMF: mycophenolate mofetil.
