# Supplementary material for: Humoral Response to mRNA-1273 SARS-CoV-2 Vaccine in Peritoneal Dialysis Patients: Is Boostering After Six Months Adequate?
Source: Front Med (Lausanne). 2022 Jun 24;9:905798. doi: 10.3389/fmed.2022.905798 (PMC9263093; doi:10.3389/fmed.2022.905798)
Supplement: Supplementary file 1 [file Data_Sheet_1.PDF]

Supplementary Figure 1:

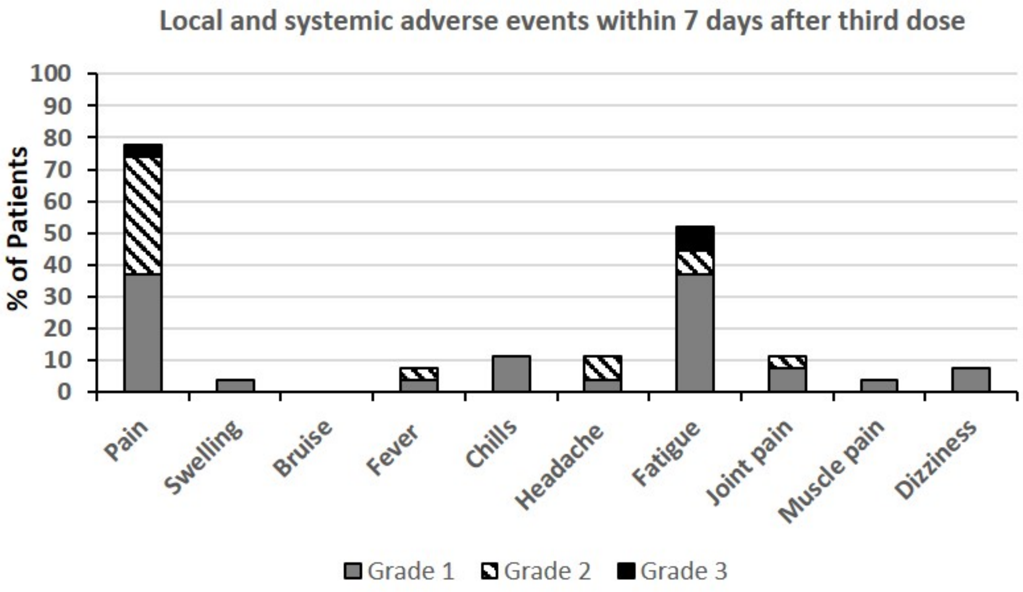

Supplementary Figure 1: Local and systemic adverse events (AEs) occurring within one week after the third dose of mRNA-1273 vaccine. The most common AEs were pain at the injection site (77.8%) and fatigue (51.9%). All reported AEs were graded as mild, moderate or severe. No hospitalizations were reported. The AEs were recorded using a standardized survey and the patients were asked to grade them using a scale from 0= no event to 4= hospitalization; grade 1= mild, does not affect daily activities; grade 2= moderate, interferes with activities of daily living; grade 3= severe, interrupts usual activities of daily living.

Supplementary Table 1:

Effects of covariates on the antibody titer after mRNA-1273 vaccine

	Coefficient	F value	p value
Age	-0.026	2.51	0.12
Gender	-0.587	1.39	0.24
Albumin	0.098	2.09	0.16
Dialysis vintage	-0.013	1.39	0.24
Vitamin D	-0.018	3.40	0.07
Ferritin	0.0005	0.61	0.44
GFR	0.136	5.82	**0.02**
Davies Comorbidity Score	-0.446	4.81	**0.03**

GFR, glomerular filtration rate
